# Suppression law of quantum states in a 3D photonic fast Fourier transform chip

**DOI:** 10.1038/ncomms10469

**Published:** 2016-02-04

**Authors:** Andrea Crespi, Roberto Osellame, Roberta Ramponi, Marco Bentivegna, Fulvio Flamini, Nicolò Spagnolo, Niko Viggianiello, Luca Innocenti, Paolo Mataloni, Fabio Sciarrino

**Affiliations:** 1 Istituto di Fotonica e Nanotecnologie, Consiglio Nazionale delle Ricerche (IFN-CNR), Piazza Leonardo da Vinci, 32, I-20133 Milano, Italy; 2 Dipartimento di Fisica, Politecnico di Milano, Piazza Leonardo da Vinci, 32, I-20133 Milano, Italy; 3 Dipartimento di Fisica, Sapienza Università di Roma, Piazzale Aldo Moro 5, I-00185 Roma, Italy; 4 Università di Roma Tor Vergata, Via della ricerca scientifica 1, I-00133 Roma, Italy

## Abstract

The identification of phenomena able to pinpoint quantum interference is attracting large interest. Indeed, a generalization of the Hong–Ou–Mandel effect valid for any number of photons and optical modes would represent an important leap ahead both from a fundamental perspective and for practical applications, such as certification of photonic quantum devices, whose computational speedup is expected to depend critically on multi-particle interference. Quantum distinctive features have been predicted for many particles injected into multimode interferometers implementing the Fourier transform over the optical modes. Here we develop a scalable approach for the implementation of the fast Fourier transform algorithm using three-dimensional photonic integrated interferometers, fabricated via femtosecond laser writing technique. We observe the suppression law for a large number of output states with four- and eight-mode optical circuits: the experimental results demonstrate genuine quantum interference between the injected photons, thus offering a powerful tool for diagnostic of photonic platforms.

## Abstract (web summary)

Computational speedup in photonic quantum devices depends on multi-particle interference, which must be certified through known benchmark algorithms. Here, to this end, the authors develop a scalable approach for the implementation of the fast Fourier transform algorithm in 3D photonic integrated interferometers.

## Graphical abstract

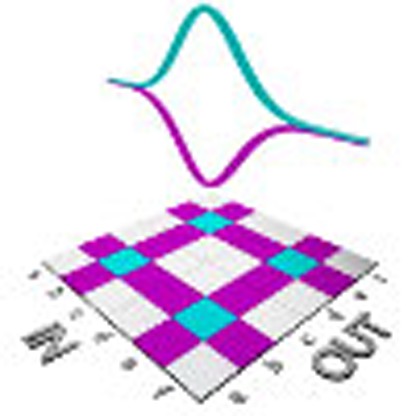

The amplitude interference between wavefunctions corresponding to indistinguishable particles lies at the very heart of quantum mechanics. Right after the introduction of laser amplification, the availability of strong coherent pulses allowed to test interference between different light pulses^1^,^2^, while generation of pairs of identical photons through parametric fluorescence^3^ led subsequently to the milestone experiment of Hong *et al.*^4^,^5^,^6^,^7^,^8^. Later on, photonic platforms have been demonstrated to be in principle capable to perform universal quantum computing^9^.

Recently, multi-particle interference effects of many photons in large interferometers are attracting a strong interest, as they should be able to show unprecedented evidences of the superior quantum computational power compared with that of classical devices^10^,^11^,^12^. The main example is given by the boson sampling^13^ computational problem, which consists in sampling from the probability distribution given by the permanents of the *n* × *n* submatrices of a given Haar random unitary. The problem is computationally hard (in *n*) for a classical computer, since calculating the permanent of a complex-valued matrix is a #P-hard problem. However, sampling from the output distribution can be efficiently achieved by letting *n* indistinguishable photons evolve through an optical interferometer implementing the unitary transformation in the Fock space, and by detecting output states with an array of single-photon detectors. The chance to provide evidences of a post-classical computation with this relatively simple set-up has triggered a large experimental effort, leading to small-scale implementations^14^,^15^,^16^,^17^,^18^,^19^,^20^, as well as theoretical analyses on the effects of experimental imperfections^21^,^22^ and on possible implementations including alternative schemes^23^,^24^.

In the context of searching for experimental evidences against the extended Church–Turing thesis, a boson sampling experiment poses a problem of certification of the result's correctness in the computationally hard regime^25^. The very complexity of the boson-sampling computational problem precludes the use of a brute-force approach, that is, calculating the expected probability distribution at the output and comparing it with the collected data. Efficient statistical techniques able to rule out trivial alternative distributions have been proposed^26^ and tested^18^,^19^, but the need for more stringent tests able to rule out less trivial distributions has led, and continues to encourage, additional research efforts in this direction.

In particular, an efficient test able to confirm true *n*-photon interference in a multimode device has been recently proposed^27^. The protocol is based on the use of an interferometer implementing the transformation described by the *n*^*p*^-dimensional Fourier matrix, with *p* being any integer. When feeding this device with multi-photon states of a specific symmetry, suppression of many output configurations is observed^28^, due to granular^27^ many-particle interference. This effect is able to rule out alternative models requiring only coarse-grained features like the ones present in Bose–Einstein condensates^29^,^30^,^31^. Indeed, the implications of this effect go well beyond the certification of boson sampling devices. As a generalization of the two-photon/two-modes Hong–Ou–Mandel (HOM) effect, the suppression law, also named Zero-Transmission law^28^, is important at a fundamental level, while at the practical level it could be used as a diagnostic tool for a wide range of photonic platforms^27^,^32^,^33^. During the review process of this work, the implementation of a discrete Fourier transform circuit in a fully reconfigurable chip has been reported^34^. The Zero-Transmission law for three-photon no-bunching events has been demonstrated in this planar six-mode interferometer.

In this article, we report the experimental observation of the recent theoretically proposed^27^ suppression law for Fourier matrices, and its use to validate quantum many-body interference against alternative non-trivial hypotheses resulting in similar output probability distributions. The Fourier matrices have been implemented with an efficient and reliable approach by exploiting the quantum version of the fast Fourier transform (qFFT), an algorithm developed by Barak and Ben-Aryeh^35^ to optimize the number of optical elements required to build the Fourier transform over the optical modes. Here we implement the qFFT on photonic integrated interferometers by exploiting the three-dimensional (3D) capabilities of femtosecond laser writing^36^,^37^, which makes it possible to fabricate waveguides arranged in 3D structures with arbitrary layouts^38^,^39^,^40^, by adopting an architecture scalable to a larger number of modes. The observations have been carried out with two-photon Fock states injected into four-mode and eight-mode qFFT interferometers. The peculiar behaviour of Fock states compared with other kinds of states is investigated, showing in principle the validity of the certification protocol for the identification of true granular *n*-particle interference, which is the source of a rich landscape of quantum effects such as the computational complexity of boson sampling.

## Results

### Suppression law in Fourier transform matrices

As a generalization of the HOM effect, it has been pointed out that quantum interference effects in multimode interferometers may determine suppression of a large fraction of the output configurations^28^,^31^,^41^, depending on the specific unitary transformation being implemented and on the symmetry of the input state. In particular^28^,^31^, let us consider a cyclic input, that is, an *n*-photon Fock state over *m*=*n*^*p*^ modes (for some integer *p*) where the occupied modes 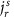 are determined by the rule 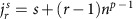, with *r*=1, …, *n* and *s*=1, …, *n*^*p*−1^. The index *s* takes into account the fact that there are *n*^*p*−1^ possible *n*-photon arrangements with periodicity *n*^*p*−1^, which simply differ by a translation of the occupational mode labels. For example, for *n*=2 and *m*=4 there are 2^1^=2 possible cyclic states, (1,0,1,0) and (0,1,0,1), while for *n*=2 and *m*=8 there are 2^2^=4 possible (collision-free) cyclic inputs, that is, the states (1,0,0,0,1,0,0,0), (0,1,0,0,0,1,0,0), (0,0,1,0,0,0,1,0) and (0,0,0,1,0,0,0,1).

We consider the evolution of such states through an interferometer implementing the transformation described by the Fourier matrix


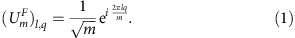


**Figure 1:**
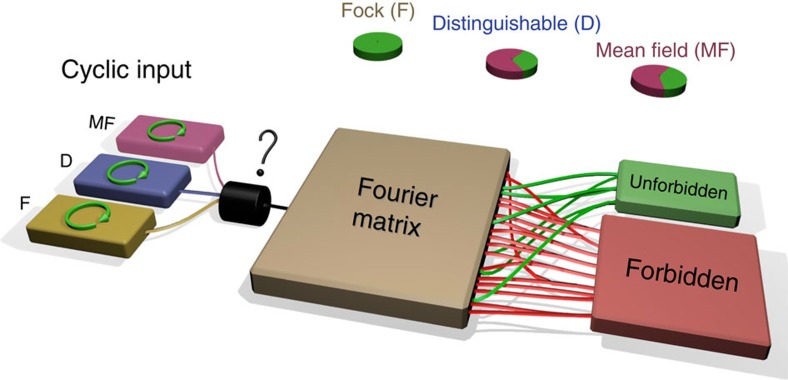
Suppression law for Fock states in a Fourier interferometer. Conceptual scheme of the protocol: the possible configurations of *n* photons at the output of an *m*-mode interferometer can be divided into two categories, unforbidden and forbidden, depending on whether they satisfy or not the suppression condition (2), respectively. The pie charts show the expected output statistics with different classes of particles, where green and red areas represent events with unforbidden and forbidden outputs, respectively. The injection of a cyclic Fock state (beige box) in an *m*-mode Fourier interferometer results in total suppression of forbidden output states. Cyclic states with distinguishable particles (blue box) show no suppression, so that each output combination is equally likely to occur. A mean field state (purple box), which reproduces some of the features of bosonic statistics, shows suppression with highly reduced contrast. Therefore, with a cyclic input the *m*-mode Fourier interferometer is able to discriminate, through the measurement of degree of violation 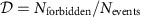, which of these three hypotheses the input state belongs to.

Such evolution results in the suppression of all output configurations not fulfilling the equation


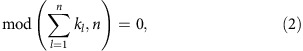


where *k*_*l*_ is the output mode of the *l*^*th*^ photon. An interesting application of suppression laws is to certify the presence of true many-body granular interference during the evolution in the interferometer, ruling out alternative hypotheses which would result in similar output probability distributions. In particular, in the case of Fourier matrices, the observation of the suppression law (2) allows to certify that the sampled output distribution is not produced by either distinguishable particles or a mean field state (MF)^27^. The latter is defined as a single-particle state 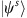 of the form


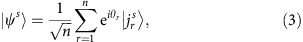


with a random set of phases *θ*_*r*_ for each state, being 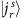 a single-particle state occupying input mode 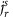. This state reproduces macroscopic interference effects, such as bunching or bosonic clouding^19^, and cannot be distinguished from true multi-particle interference with criteria based on these features. Since it is possible to efficiently simulate the evolution of a MF with a classical algorithm^27^, it is of fundamental importance to assess the ability of a validation scheme to discriminate such a state in the context of an untrusted party claiming to perform a boson sampling experiment. Hence, the MF represents an optimal test bed for the certification protocol based on the suppression law (see Fig. 1).

It is possible to quantify the degree of violation 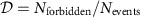 of the suppression law as the number of observed events in forbidden output states divided by the total number of events^27^. If a Fock state is injected in a Fourier interferometer, a violation 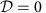 would be observed. In the case of distinguishable photons there is no suppression law, and the violation would be simply the fraction of suppressed outputs, each one weighted with the number of possible arrangements of the *n* distinguishable particles in that output combination. In the case of two-photon states, the weighting factor is 2 for collision-free outputs and 1 otherwise, and a degree of violation of 1/2 is expected (see Section ‘Observation of the suppression law'). In contrast, in the case of two-photon MF, bunching effects occur leading to an expected degree of violation of half the weighted fraction of suppressed outputs (1/4 for two-photon MF). It has been shown that the fraction of forbidden outputs is always large^31^. Hence, a comparison of the observed value of 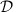 with the expected one represents an efficient way, in terms of necessary experimental runs, to discriminate between Fock states, distinguishable particles states and MFs.

### Realization of 3D qFFT interferometers

Let us now introduce our experimental implementation of the qFFT. The general method to realize an arbitrary unitary transformation using linear optics was introduced by Reck *et al.*^42^, who provided a decomposition of a unitary of dimension *m* as a sequence of *m*(*m*–1)/2 beam splitters and phase shifters. However, in the special case of Fourier matrices a more efficient method has been proposed^35^,^43^, which takes advantage of their symmetries to significantly reduce the number of linear optical elements required. On the basis of the classical algorithm of Cooley and Tukey^44^, who first introduced the fast Fourier transform algorithm as a more efficient way to calculate the discrete Fourier transform, Barak and Ben-Aryeh developed a quantum analogue in the linear optics domain, leading to the concept of qFFT. This approach, valid for 2^*p*^-dimensional Fourier matrices, requires only (*m*/2)log*m* beam splitters and phase shifters, to be compared with the *O*(*m*^2^) elements needed for the more general Reck decomposition, thus enhancing the compactness and scalability of the platform for a more reliable experimental realization. The overall linear transformation on the optical modes implemented by the qFFT circuit is naturally equivalent to the transformation described by the Fourier matrix, hence 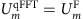.

**Figure 2:**
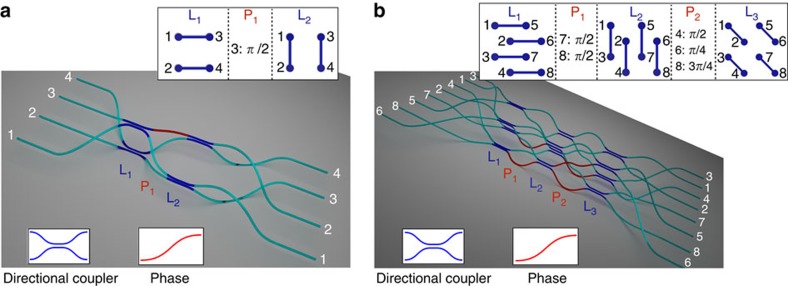
Schematic representation of the structure of the integrated devices. Internal structure of the four-mode (**a**) and eight-mode (**b**) integrated interferometers implementing the qFFT over the optical modes. In the eight-mode case, the Barak and Ben-Aryeh algorithm requires an additional relabelling of the output modes (not shown in the figure), namely 2↔5 and 4↔7, to obtain the effective Fourier transformation. The mode arrangement has been chosen in a way to minimize bending losses. The insets show the actual disposition of the waveguides in the cross-section of the devices. The modes coupled together in each step (L_*i*_) of the interferometer are joined by segments. The implemented phase shifts in each step (P_*i*_) are also indicated.

Here we introduce a new methodology for an integrated implementation of the qFFT, which exploits the 3D capabilities of the femtosecond laser writing technique. The sequential structure arising from the decomposition of the *m*-dimensional Fourier matrix using the Barak and Ben-Aryeh algorithm is reproduced by the consecutive layers shown in Fig. 2. The complex arrangement of pairwise interactions necessary for the qFFT method cannot be easily implemented using a planar architecture. However, femtosecond laser writing technique allows to overcome this issue exploiting the third dimension, arranging the waveguides along the bidimensional sections of the integrated chip.

The strategy can be outlined as follows (see also Supplementary Note 1): the 2^*p*^ modes are ideally placed on the vertices of a *p*-dimensional hypercube; in each step of the algorithm the vertices connected by parallel edges having one specific direction are made to interact by a two-mode Hadamard transformation, with proper phase terms. An optical interferometer implementing this procedure is thus composed of log_2_*m*=*p* sections, each employing *m*/2 balanced beam splitters and phase shifters.

We fabricated waveguide interferometers realizing the Fourier matrix for *m*=4 and 8 modes in borosilicate glass chips using femtosecond laser micromachining^36^,^37^. A schematic representation of these two interferometers is given in Fig. 2. According to the scheme outlined above and by exploiting the 3D capabilities of the fabrication technique, the waveguides are placed, for what concerns the cross-section of the device, on the vertices of a two-dimensional projection of the *p*-dimensional hypercube (see also Supplementary Fig. 1). 3D directional couplers, with proper interaction length and distance to achieve a balanced splitting, connect in each step the required vertices. The insets of Fig. 2 show, at each step *i*, which modes are connected by directional couplers (L_*i*_) and the amount of phase shift that needs to be introduced in specific modes (P_*i*_). Phase shifters, where needed, are implemented by geometrical deformation of the connecting S-bends. Fan-in and fan-out sections at the input and output of the devices allows interfacing with 127 μm spaced single-mode fibre arrays. Note that in our device geometry, in each step, the vertices to be connected are all at the same relative distance. This means that, unless geometric deformations are designed where needed, light travelling in different modes does not acquire undesired phase delays. It is worth noting that the geometric construction here developed is scalable to an arbitrary number of modes with a number of elements increasing as *m*log_2_*m*.

### One- and two-photon measurements

**Figure 3:**
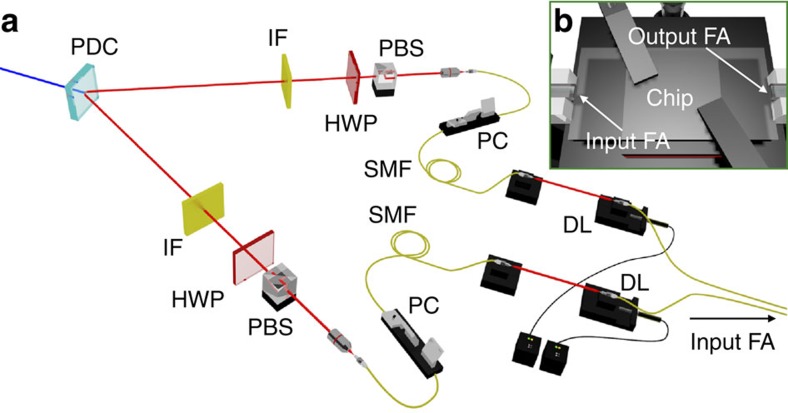
Experimental apparatus for input state preparation. (**a**) The photon source (IF, HWP, PBS, PC, PDC, DL and SMF). (**b**) Photon injection (extraction) before (after) the evolution through the interferometer. DL, delay lines with motorized stages; FA, fibre array; HWP, half-wave plate; IF, interferential filter; PBS, polarizing beam splitter; PC, polarization compensator; PDC, parametric downconversion; SMF, single-mode fibre.

The two implemented interferometers of *m*=4 and 8 modes are fed with single- and two-photon states. The experimental set-up, preparing a biphoton wave packet to be injected into the devices, is shown in Fig. 3. Further details on the photon generation and detection scheme are described in the Methods section. To test the validity of the suppression law, we measured the number of coincidences at each forbidden output combination injecting cyclic inputs with two indistinguishable photons. The degree of violation 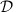 of the suppression law could simply be evaluated with a counting experiment. Alternatively, the same quantity 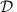 can be expressed as a function of single-photon input–output probabilities and of the HOM visibilities, defined as


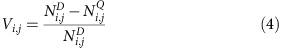


where 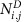 is the number of detected coincidences for distinguishable photons and 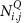 for indistinguishable photons. The subscripts (*i*,*j*) are the indexes of the two output modes, for a given input state. The degree of violation can therefore be expressed as


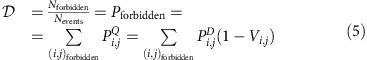


where 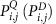 are the probabilities of having photons in the outputs *i*,*j* in the case of indistinguishable (distinguishable) particles. Here 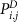 can be obtained from single-particle probabilities. The visibilities are measured by recording the number of coincidences for each output combination as a function of the temporal delay between the two injected photons.

**Figure 4:**
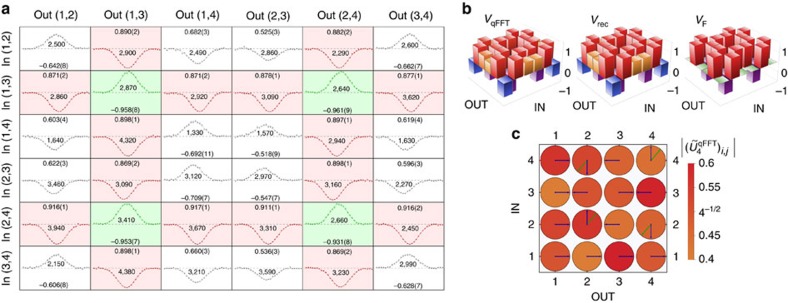
Suppression law in a four-mode qFFT integrated chip. (**a**) Complete set of 36 measured coincidence patterns (raw experimental data) for all input–output combinations in the four-mode chip. For each input–output combination, the measured coincidence pattern as a function of the time delay is shown (points: experimental data, lines: best-fit curves). Cyclic inputs (1,3) and (2,4) exhibit enhancement (green) and suppression (red) on cyclic and non-cyclic outputs, respectively. For all points, error bars are due to the Poissonian statistics of the events. In each subplot the measured visibility with corresponding error and the sample size are reported. For each visibility, the error is obtained through a Monte Carlo simulation by averaging over 3,000 simulated data sets. In each subplot the zero level coincides with the baseline, while a dashed line represents the number of coincidence events in the distinguishable limit. (**b**) HOM visibilities for all 36 input–output configurations. (left to right) Experimental measured visibilities (*V*_qFFT_, obtained from raw experimental data), visibilities calculated from the reconstructed unitary (*V*_rec_), and visibilities calculated from the theoretical unitary (*V*_F_). (**c**) Representation of the reconstructed experimental transformation 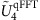, and comparison with 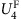. Coloured disks represent the moduli of the reconstructed matrix elements (all equal to 4^−1/2^ for 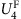). Arrows represent the phases of the unitary matrix elements (green: reconstructed unitary, blue: Fourier matrix).

For the four-mode device, we measured the full set of 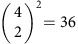 collision-free input–output combinations, that is, where the two photons exit from different output ports. These contributions have been measured by recording the number of coincidences for each combination of two outputs as a function of the temporal delay between the two input photons. Because of the law given by equation (2), we expect to observe four suppressed outcomes (over six possible output combinations) for the two cyclic input states (1,3) and (2,4). Since distinguishable photons exhibit no interference, HOM dips in the coincidence patterns are expected for the suppressed output states. Conversely, peaks are expected in the non-suppressed output combinations. The experimental results are shown in Fig. 4a, where the expected pattern of four suppressions and two enhancements is reported, with average visibilities of 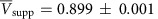 and 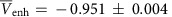 for suppression and enhancement, respectively.

For the cyclic inputs, we also measured the interference patterns for the output contributions where the two photons exit from the same mode. These terms have been measured by inserting an additional symmetric beam splitter on each output mode, and by connecting each of its two outputs to a single-photon detector. These cases correspond to a full-bunching scenario with *n*=2, and a HOM peak with *V*=−1 visibility is expected independently from the input state and from the unitary operation^45^. This feature has been observed for the tested inputs, where an average visibility of 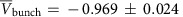 has been obtained over all full-bunching combinations. Note that the measured two-mode correlation matrix is not compatible with classical light (see Supplementary Note 4).

The existence of a general rule for the prediction of suppressed output combinations when injecting a cyclic Fock state in a Fourier interferometer is due to the intrinsic symmetry of the problem, as opposed to the general boson sampling scenario^13^. Suppressed outputs for non-cyclic inputs can be predicted by calculating the permanent of the submatrix given by the intersection of the columns and rows of *U*^F^ corresponding to the occupied input and output modes, respectively. The complete set of measured dips and peaks is shown in Fig. 4a, highlighting the symmetry in the Fourier transform interference pattern. The injection of the non-cyclic input states has been employed for the complete reconstruction of the chip action 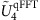, using a data set statistically independent from the one adopted to observe the suppression law. The adopted reconstruction algorithm, which exploits knowledge on the internal structure of the interferometers (specified in Fig. 2), works in two steps. In a first step, the power-splitting ratios measured with classical light are employed to extrapolate the transmissivities of the directional couplers. In a second step, the two-photon visibilities for the non-cyclic inputs are used to retrieve the values of the fabrication phases. In both steps the parameters are obtained by minimizing a suitable *χ*^2^ function. The results are shown in Fig. 4c. The fidelity between the reconstructed unitary 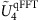 and the theoretical Fourier transform 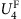 is 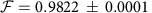, thus confirming the high quality of the fabrication process. The error in the estimation of the fidelity is obtained through a Monte Carlo simulation, properly accounting for the degree of distinguishability of the photons with a rescaling factor in the visibilities.

**Figure 5:**
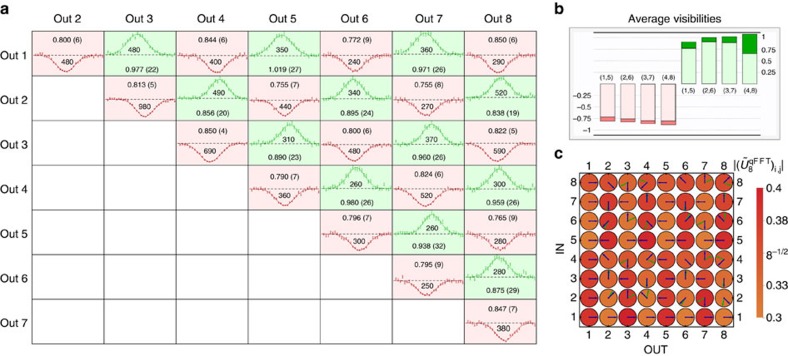
Suppression law in a eight-mode qFFT integrated chip. (**a**) Set of 28 measured coincidence patterns (raw experimental data), corresponding to all collision-free output combinations for the input (2,6) of the eight-mode interferometer. For each output combination, the measured coincidence pattern as a function of the time delay is shown (points: experimental data, lines: best-fit curves). Red or green backgrounds correspond to dips and peaks, respectively. For all points, error bars are due to the Poissonian statistics of the events. In each subplot the measured visibility with corresponding error and the sample size are reported. For each visibility, the error is obtained through a Monte Carlo simulation by averaging over 3,000 simulated data sets. In each subplot the zero level coincides with the baseline, while a dashed line represents the number of coincidence events in the distinguishable limit. (**b**) Average visibilities of dips (red bars) and peaks (green bars) observed for the four collision-free cyclic inputs [(1,5), (2,6), (3,7), (4,8)]. Darker regions correspond to error bars of ±1 s.d. (**c**) Representation of the reconstructed experimental transformation 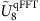, and comparison with 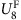. Coloured disks represent the moduli of the reconstructed matrix elements (all equal to 8^−1/2^ for 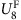). Arrows represent the phases of the unitary matrix elements (green: reconstructed unitary, blue: Fourier matrix).

For the eight-mode chip we recorded all the 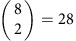 two-photon coincidence patterns, as a function of the relative delay between the input photons, for each of the four collision-free cyclic inputs and for one non-cyclic input. The reconstruction of the actual unitary transformation 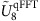 implemented has been performed with the same algorithm of the four-modes, by using the power-splitting ratios measured with classical light and the two-photon visibilities for one non-cyclic input. The latter has been chosen in a way to maximize the sensitivity of the measurements with respect to the five fabrication phases. The results are shown in Fig. 5. The fidelity between the reconstructed unitary 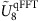 and the ideal eight-mode Fourier transform 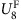 is 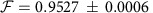. More details on the reconstruction algorithm can be found in the Supplementary Note 3.

### Observation of the suppression law

The suppression of events which do not satisfy equation (2) is fulfilled only when two perfectly indistinguishable photons are injected in a cyclic input of a perfect Fourier interferometer. In such a case, we would have the suppression of all output states whose sum of the indexes corresponding to the occupied modes is odd. For the four-mode (eight-mode) interferometer, this corresponds to four (16) suppressed and two (12) non-suppressed collision-free outputs (each one given by two possible arrangements of the two distinguishable photons), plus four (8) terms with two photons in the same output, each one corresponding to a single possible two-photon path.

The expected violation for distinguishable particles can be obtained from classical considerations. Let us consider the case with *n*=2. The two distinguishable photons evolve independently from each other, and the output distribution is obtained by classically mixing single-particle probabilities. All collision-free terms are equally likely to occur with probability *q*=2/*m*^2^, while full-bunching events occur with probability *q*′=*q*/2=1/*m*^2^. The degree of violation 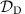 can then be obtained by multiplying the probability *q* by the number of forbidden output combinations. As a result, we expect a violation degree of 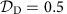 for distinguishable two-photon states. The evaluation of the expected value for a MF state, which is due to single-particle bosonic statistic effects, requires different calculations^27^. It can be shown that for *n*=2 the degree of violation is 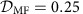.

**Figure 6:**
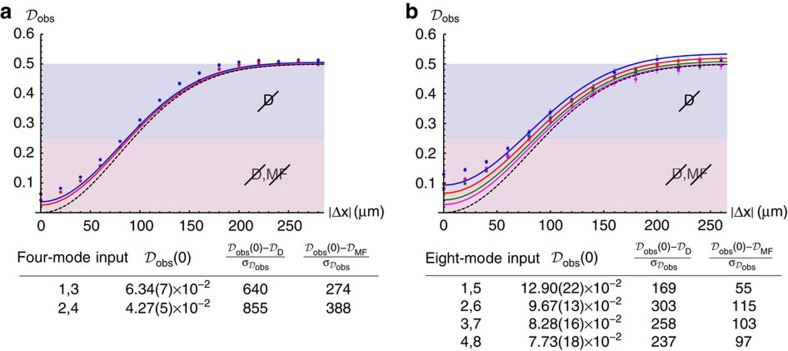
Measured violations. Observed violations 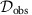 as a function of the path difference |Δ*x*|=*c*|Δ*τ*| between the two photons. Blue shaded regions in the plots correspond to the cases where the hypothesis of distinguishable particles can be ruled out. Red regions correspond to the cases when both the hypotheses of distinguishable particles and mean field state can be ruled out, and true two-particle interference is present. (**a**) Data for the four-mode interferometer. Blue points: input (1,3). Red points: input (2,4). Blue solid line: theoretical prediction for input (1,3). Red solid line: theoretical prediction for input (2,4). Black dashed line: theoretical prediction for a Fourier matrix. (**b**) Data for the eight-mode interferometer. Blue points: input (1,5). Red points: input (2,6). Green points: input (3,7). Magenta points: input (4,8). Coloured solid lines: corresponding theoretical predictions for the different inputs. Black dashed line: theoretical prediction for a Fourier matrix. Tables: violations 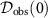 at Δ*x*=0 and discrepancies (in sigmas) with the expected values for distinguishable particles (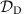) and MFs (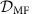), for the cyclic inputs of the two interferometers. 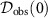 are calculated following formula (5), while expected values for the other two cases are 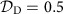 and 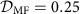. Error bars in all experimental quantities are due to the Poissonian statistics of measured events. All theoretical predictions in solid lines are calculated from the reconstructed unitaries, obtained from different sets of experimental data to ensure statistical independence. See Supplementary Note 2 for the modelling of imperfect state preparation.

For each of the cyclic input, we have evaluated here the violation degree 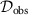 resulting from collected data. By measuring the coincidence pattern as a function of the path difference Δ*x*=*c*Δ*τ* between the two photons, and thus by tuning their degree of distinguishability, we could address the transition from distinguishable to indistinguishable particles. The value of 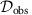 as a function of Δ*x* has been obtained as 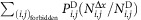, where 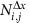 and 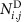 are the number of measured coincidences for a given value of Δ*x* and for distinguishable particles respectively. Two different regions can be identified. For intermediate values of Δ*x* with respect to the coherence length of the photons, the measured data fall below the threshold 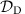, and hence the hypothesis of distinguishable particles can be ruled out. Then, for smaller values of the path difference up to Δ*x*→0, true two-photon interference can be certified since both hypothesis of distinguishable particles and MF state can be ruled out. The maximum violation occurring at Δ*x*=0 delay can be evaluated using equation (5). The experimental results retrieved from the protocol are shown in the tables of Fig. 6, in which we compare the values 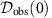 with the expected values for distinguishable particles 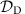 and for a MF state 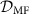. As shown for our implementation, the robustness of the protocol is ensured by the high number of s.d. separating the values in each comparison, thus unambiguously confirming the success of the certification protocol. In conclusion, the alternative hypotheses of distinguishable particles and of a MF state can be ruled out for all experiments.

## Discussion

We have reported on the experimental observation of the suppression law on specific output combinations of a Fourier transformation due to quantum interference between photons. The observation of the suppression effect allowed us to rule out alternative hypotheses to the Fock state. The use of a novel implementation architecture, enabled by the 3D capabilities of femtosecond laser micromachining, extends the scalability of this technique to larger systems with lower experimental effort with respect to other techniques. While the presented architecture is designed to implement a Fourier matrix for a number of modes equal to *m*=2^*p*^, a generalization of the approach can be obtained by adopting a building block different from the beam splitter. For devices of odd dimension, for instance, such a tool can be provided by the tritter transformation^39^. At the same time, the universality of a generalized HOM effect with an arbitrary number of particles and modes is expected to make it a pivotal tool in the diagnostic and certification of quantum photonic platforms. Boson sampling represents a key example, since the scalability of the technique is expected to allow efficient certification of devices outperforming their classical counterparts. An interesting open problem is whether the computational hardness of boson sampling is maintained if the Haar-randomness condition is relaxed^46^, and thus which is the minimal interferometer architecture required for an evidence of post-classical computation.

Fourier matrices can find application in different contexts. For instance, multiport beam splitters described by the Fourier matrix can be employed as building blocks for multiarm interferometers, which can be adopted for quantum-enhanced single and multiphase estimation protocols^47^. This would also allow the measurement of phase gradients with precision lower than the shot-noise limit^48^. Other fields where Fourier matrices are relevant include quantum communication scenarios^49^, observation of two-photon correlations as a function of geometric phase^50^, fundamental quantum information theory including mutually unbiased bases^51^, as well as entanglement generation^52^.

## Methods

### Waveguide device fabrication

Waveguide interferometers are fabricated in EAGLE2000 (Corning Inc.) glass chips. To inscribe the waveguides, laser pulses with 300 fs duration, 220 nJ energy and 1 MHz repetition rate from an Yb:KYW cavity dumped oscillator (wavelength 1,030 nm) are focused in the bulk of the glass, using a 0.6 NA microscope objective. The average depth of the waveguides, in the 3D interferometric structure, is 170 μm under the sample surface. The fabricated waveguides yield single-mode behaviour at 800 nm wavelength, with about 0.5 dB cm^−1^ propagation losses. The central part of the 3D interferometer, which includes all the relevant couplers, have a cross-section of about 50 μm × 50 μm (95 μm × 95 μm) for a length of 9.0 mm (14.7 mm) in the four-(eight-)modes case. The length of each fan-in and fan-out section, needed to bring the waveguides at 127 μm relative distance, is 7.8 mm (13.2 mm).

### Photon generation and manipulation

The generation of two-photon states is performed by pumping a 2-mm long BBO crystal with a 392.5 nm wavelength Ti:Sa pulsed laser, with average power of 750 mW, which generates photons at 785 nm with a type II parametric downconversion process. The two photons are spectrally filtered by means of 3 nm interferential filters, and coupled into single-mode fibres. The indistinguishability of the photons is then ensured by a polarization compensation stage, and by propagation through independent delay lines (used to adjust the degree of temporal distinguishability) before injection within the interferometer via a single-mode fibre array. After the evolution through the integrated devices, photons are collected via a multimode fibre array. The detection system consists of four (8) single-photon avalanche photodiodes used for the four- (eight-) modes chip. An electronic data acquisition system allowed us to detect coincidences between all pairs of output modes. Typical coincidence rates for each collision-free output combination with distinguishable photons amounted to ∼70–80 Hz (for the four-mode chip) and ∼10–20 Hz (for the eight-mode chip).

## Additional information

**How to cite this article:** Crespi, A. *et al.* Suppression law of quantum states in a 3D photonic fast Fourier transform chip. *Nat. Commun.* 7:10469 doi: 10.1038/ncomms10469 (2016).

## Supplementary Material

Supplementary Information Supplementary Figures 1-3, Supplementary Notes 1-4 and Supplementary References.
