# Supplementary material for: Suppression law of quantum states in a 3D photonic fast Fourier transform chip
Source: Nat Commun. 2016 Feb 4;7:10469. doi: 10.1038/ncomms10469 (PMC4742850; doi:10.1038/ncomms10469)
Supplement: Supplementary Information — Supplementary Figures 1-3, Supplementary Notes 1-4 and Supplementary References. [file ncomms10469-s1.pdf]

**Dimension**	**Planar projection of the hypercube**
$p = 1$	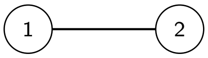
$p = 2$	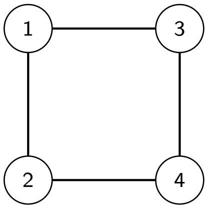
$p = 3$	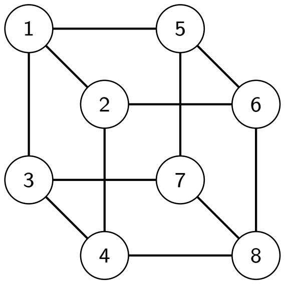
$p = 4$	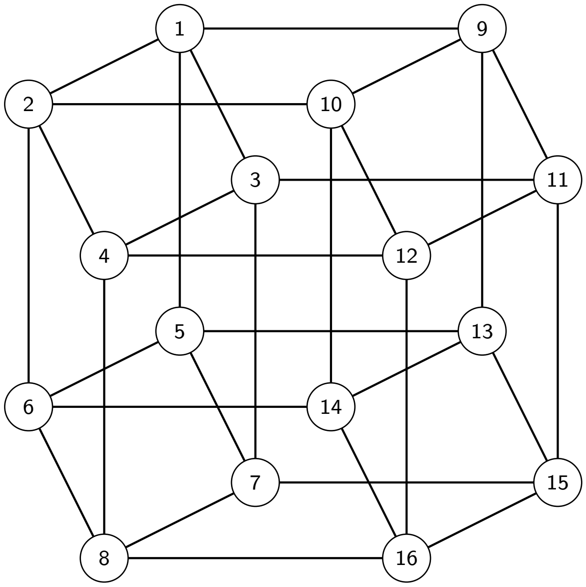

Supplementary Figure 1. **Planar projections of hypercubes of dimension p up to 4.** The numbered vertices correspond to the possible position of the waveguides in the cross-section of a 3D interferometer which implements the quantum Fast Fourier Transform. Each connection between the vertices corresponds to a directional coupler in a given section of the interferometer.

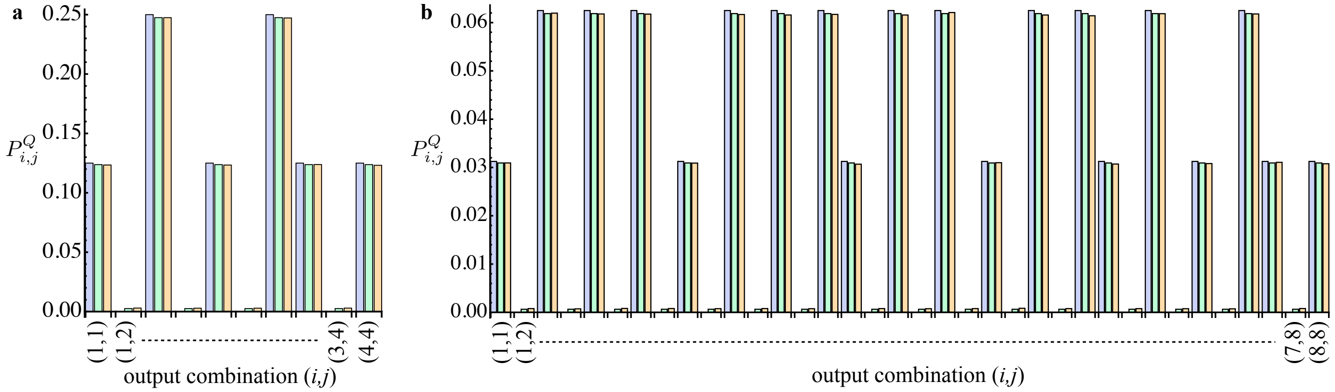

Supplementary Figure 2. **Numerical analysis of noise contributions due to imperfect state preparations**. Comparison of the output probability distributions for a cyclic input with an ideal two-photon state (blue bars), a two-photon state with non-unit overlap (green bars), and an input state with multiphoton emission terms (orange bars). The unitary adopted is the Fourier matrix. The parameters used in the different cases are described in the text. The distributions for the multiphoton emission terms are obtained from a numerical simulation of the process with 10^6 events. (**a**) 4-mode interferometer and (**b**) 8-mode interferometer.

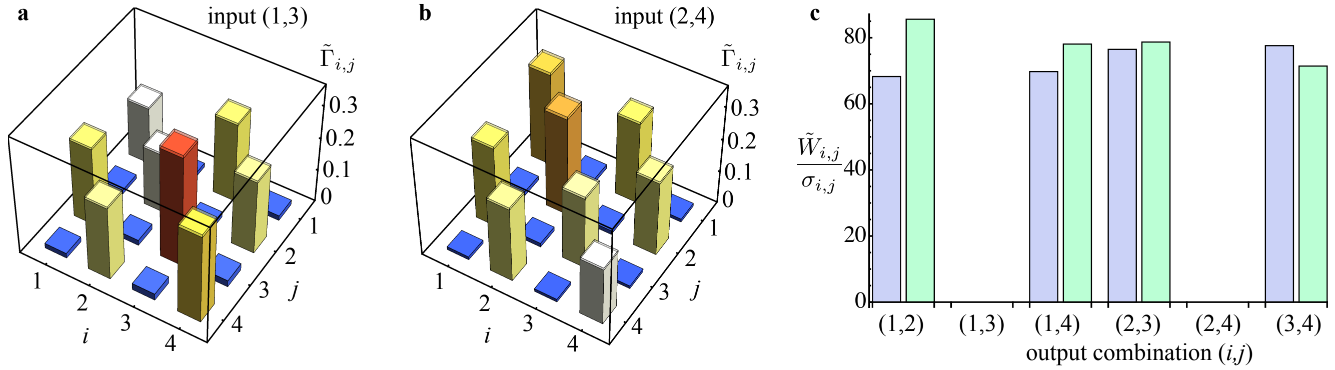

Supplementary Figure 3. **Discrimination of two-photon data with respect to classical light.** Two-photon correlations $\tilde{\Gamma}_{i,j}$ obtained from experimental data for the two cyclic inputs in the 4-mode interferometer: (**a**) input modes (1,3) and (**b**) input modes (2,4). (**c**) Experimental results for the discrimination test against classical light with the 4-mode interferometers and two-photon input: number of standard deviation for which the parameter $\tilde{W}_{i,j}$ violates Eq. (S6) for each output combination. Blue bars: input modes (1,3). Green bars: input modes (2,4).

SUPPLEMENTARY NOTE 1: INTERFEROMETER DESIGN IN THE 2^p-MODES CASE

In the following, we will schematically discuss the procedure to design an interferometer by implementing the quantum Fast Fourier Transform (qFFT) in the general case of $m = 2^p$ modes, for some integer p.

- Each optical mode (waveguide) $k \in [1, m]$ can be numbered according to its binary representation and can be associated to the set

$$(b_1, b_2, ..., b_p)_k$$

  where $b_i = -1$ or $+1$ if the i-th bit of the binary representation of k is 0 or 1 respectively. The ordering of the b_is is intended to be from the most significant bit b_1 to the least significant bit b_p.

- The p-element vector $(b_1, b_2, ..., b_p)$ represents the coordinates of the vertices of a p-dimensional hypercube in $\mathbb{R}^p$.

- The optical qFFT algorithm [1] consists of p steps. The j-th step connects, making them interact, all the couples of modes that differ only for the j-th bit. (Proper phase terms are also added and a final relabelling of the outputs is needed, but this is not relevant here).

- The position of each waveguide in the cross-section plane can be defined by suitably projecting in two dimensions the vertices of the hypercube. Each step of the qFFT corresponds to connecting, by directional couplers, the couples of modes that have to interact in that step. In particular, such connections corresponds to edges of the hypercube with a given direction, all parallel to each other (note that the edges of a p-dimensional hypercube are placed along p possible directions).

- It is worth noting that with this layout, in each step, modes that have to be connected by couplers are all at the same relative distance and the projection of the couplers on the plane are all parallel lines. This means that such couplers can be all identical (except for deformations that may be needed for introducing phase terms) and waveguides never cross. This avoids parasitic coupling between modes that should stay separated and unwanted differences in the optical paths of the mode connections.

SUPPLEMENTARY NOTE 2: MULTIPHOTON EMISSION TERMS

The main sources of experimental imperfections in the state preparation are due to multi-photon emission terms occurring in SPDC processes and to non-unitary indistinguishability of the two photons. Here we perform a numerical analysis on how these two effects lead to the presence of added noise in the suppressed contributions, making harder to violate the validation criteria.

The role of non-unitary indistinguishability in multiphoton interference, and in particular in Boson Sampling, has been discussed in several works [3-5]. In our work, we model this effect by considering an input state of the form:

$$\rho = \alpha|1, 1\rangle\langle 1, 1| + (1 - \alpha)|1_a, 1_b\rangle\langle 1_a, 1_b|, \tag{S1}$$

where α represents the degree of overlap of the two-photon state, and $|1_a, 1_b\rangle$ stands for two distinguishable photons. Hence, the output probability distribution is the weighted mixture of the distributions $P^Q_{i,j}$ (indistinguishable photons) and $P^D_{i,j}$ (distinguishable photons).

Multiphoton emission is due to the probabilistic nature of the photon-pair generation in SPDC sources. Being g the nonlinear gain of the process, the output of an SPDC source in the low gain limit can be written as

$$|\psi\rangle \sim |0, 0\rangle + g|1, 1\rangle + g^2|2, 2\rangle. \tag{S2}$$

Multiphoton emission then leads to the probabilistic injection of input states different from the expected one. Since losses are present in the apparatus and non photon-number resolving detectors are employed, these multiphoton events generated by multiphoton emission cannot be discriminated from the events generated by the correct input state. Furthermore photons belonging to different pairs can present a lower value of distinguishability than photons belonging to the same pair. Note that in the discussed configuration the lower is the distinguishability between different pairs, the higher is the noise contribution due to multiphoton emission. Indeed, for full indistinguishability between different photon pairs the noisy state injected in the cyclic inputs presents the same symmetry of the correct input $|1, 1\rangle$, and thus some suppressed events are present reducing the amount of additional noise.

In Supplementary Figure 2 we show the results of numerical calculations for the two noise effects for values of α (non-unitary overlap), g (non linear gain), and losses corresponding to our experimental implementation. In our case $\alpha \sim 0.98$ (estimated from the visibility of an Hong-Ou-Mandel experiment in a 50/50 beam-splitter), while $g \sim 0.12$ (estimated from single-photon counts and two-photon coincidences of the source). For the sake of simplicity, losses have been grouped in two main contributions (input and output). The overall input η_{in} and output η_{out} transmissivities, including also coupling efficiency of the source and detection efficiency, were in our case ($\eta_{in} \sim 0.1, \eta_{out} \sim 0.28$ for 4-mode device) and ($\eta_{in} \sim 0.085, \eta_{out} \sim 0.23$ for 8 mode device). Propagation losses in the device have been equally distributed in the two parameters (η_{in} and η_{out}). We observe that the two effects of multiphoton emission and non-unit overlap lead to similar noise contributions, that is, the observation of events in suppressed outputs. Both imperfections in our experiment can thus be modeled with the input state of Eq. (S1), by considering an effective overlap parameter $\alpha_{\text{eff}} \sim 0.95$.

SUPPLEMENTARY NOTE 3: RECONSTRUCTION OF THE DEVICES TRANSFORMATION

In order to characterize the action of the implemented devices, we adopted a reconstruction method which allows us to retrieve the internal parameters of the interferometers (directional couplers transmissivities t_i and fabrication phases ϕ_i). The reconstructed unitary transformation is then obtained from the values of the characterized internal parameters. We also considered in the process the possibility of unbalanced output losses between the different modes of the interferometers. This effect can be taken into account by modeling the device with a unitary transformation $U(\{t_i\}, \{\phi_i\})$ followed by a diagonal matrix $L(\{\eta_i\})$ with elements $\sqrt{\eta_i}$, which represent the relative output losses. Since we are dealing with relative quantities, we set $\eta_1 = 1$ as a reference. The overall transformation M is then obtained as:

$$M(\{t_i\}, \{\phi_i\}, \{\eta_i\}) = L(\{\eta_i\})\, U(\{t_i\}, \{\phi_i\}) = \begin{pmatrix} 1 & 0 & \dots & 0 \\ 0 & \sqrt{\eta_2} & \dots & 0 \\ \dots & \dots & \dots & \dots \\ 0 & 0 & \dots & \sqrt{\eta}_m \end{pmatrix} U(\{t_i\}, \{\phi_i\}) \tag{S3}$$

The internal parameters ($\{t_i\}, \{\phi_i\}$) and the output losses $\{\eta_i\}$ are obtained from a two-step procedure.

Step 1. By adopting single-photon measurements, or equivalently by measuring the power-splitting ratios with classical light, the output losses $\{\eta_i\}$ and the directional couplers transmittivities $\{t_i\}$ are retrieved by minimizing the following χ^2 function:

$$f_1 = \sum_{j=1}^{m} \sum_{k=1}^{m} \frac{[P_{j,k}(\{t_i\}, \{\eta_i\}) - \tilde{P}_{j,k}]^2}{(\Delta \tilde{P}_{j,k})^2} \tag{S4}$$

Here $\tilde{P}_{j,k}$ are the measured power-splitting ratios and $\Delta \tilde{P}_{j,k}$ are the corresponding experimental errors. Conversely $P_{j,k}(\{t_i\}, \{\eta_i\})$ are the predictions calculated from the unknown transformation, which depend on the sets $\{t_i\}$ and $\{\eta_i\}$. Note that for the internal structure of our interferometers, the power-splitting ratios do not depend on the fabrication phases $\{\phi_i\}$.

Step 2. The values of the set $\{\phi_i\}$ are retrieved by exploiting two-photon visibility measurements. More specifically, we used the visibilities of all the non-cyclic inputs (in the 4-mode case) and of a non-cyclic input (in the 8-mode case). The latter has been chosen so as to maximize the sensitivity to the fabrication phases $\{\phi_i\}$. The phases $\{\phi_i\}$ are then obtained by minimizing the following χ^2 function:

$$f_2 = \sum_{l} \frac{[\alpha V_l(\{\bar{t}_i\}, \{\phi_i\}) - \tilde{V}_l]^2}{(\Delta V_l^M)^2} \tag{S5}$$

where the sum over the index l extends over the measured input-output combinations. Here, $\{\tilde{V}_l\}$ are the measured visibilities, while α is the indistinguishability parameter of our two-photon source (see above). The visibilities $\{\tilde{V}_l\}$ adopted in the reconstruction process have been obtained by subtracting the accidental coincidences from the experimental data so as to correct for multiphoton emission of the source. Conversely, $V_l(\{\bar{t}_i\}, \{\phi_i\})$ are the theoretical predictions obtained from the unknown unitary, which are calculated for the values $\{\bar{t}_i\}$ of the transmissivities obtained from the first step. Note that two-photon visibilities do not depend on the output losses $\{\eta_i\}$.

Errors on the reconstruction process are obtained by a Monte Carlo simulation. Starting from the set of measured experimental data, we randomly simulated new sets of power-splitting ratios and two-photon data according to a Gaussian distribution, with μ and σ equal to the experimental data and errors respectively. Additionally, we inserted

a random rescaling factor on the simulated visibility to take into account the error on the indistinguishability parameter α. For each set of randomly generated data, we applied the two-step reconstruction method to obtain a new unitary. The error bars on the fidelities are then obtained by evaluating the standard deviation of the fidelity from the generated set of unitaries. The reconstruction errors on the internal parameters can be estimated simultaneously with the same approach.

The results obtained show that the unbalancement of the relative output losses is in the range $\overline{\eta}_i \in [1, 1.135]$ for the 4-mode device, and in the range $\overline{\eta}_i \in [0.864, 1.056]$ for the 8-mode device. The results for the unitary transformations are shown in the main text.

To test the validity of the approach, the unitary obtained with the two-step method has been compared with the one retrieved by applying the reconstruction algorithm of Ref. [2]. The fidelity between the reconstructed unitary transformations with the two methods is $\mathcal{F} > 0.999$, which shows that the two approaches converge to almost identical results.

For a detailed discussion on the role of imperfections in the implemented unitary in the context of multiphoton interference refer to [6-7].

SUPPLEMENTARY NOTE 4: DISCRIMINATION FROM CLASSICAL LIGHT

The measured two-photon data can be discriminated from classical light by using a test which involves both collision-free and bunching contributions [3,4]. This test is based on the two-mode correlation matrix, which is defined as $\Gamma_{i,j} = \langle a_i^\dagger a_j^\dagger a_j a_i \rangle$. For two-photon input states, this reads $\Gamma_{i,j} = (1 + \delta_{i,j}) P^Q_{i,j}$, where $P^Q_{i,j}$ is the output probability. The correlation matrix for classical light must obey the following inequality for all (i, j):

$$W_{i,j} = \frac{1}{3}\sqrt{\Gamma^{(\mathrm{cl})}_{i,i}\Gamma^{(\mathrm{cl})}_{j,j}} - \Gamma^{(\mathrm{cl})}_{i,j} < 0 \tag{S6}$$

A violation of this inequality indicates that the collected data present correlations which are stronger than those achievable with classical light.

We then applied this test to our experimental data, corresponding to the two cyclic inputs injected in the 4-mode interferometer. The experimental correlation matrix $\tilde{\Gamma}_{i,j}$, shown in Supplementary Figure 3a-b, has been retrieved from single-photon measurements and two-photon visibilities. The violation parameters $\tilde{W}_{i,j}$ and the corresponding errors $\sigma_{i,j}$ can then be obtained from $\tilde{\Gamma}_{i,j}$. We observe a violation of inequality (S6) of more than 68 standard deviations, for all suppressed contributions in both cyclic inputs (see Supplementary Figure 3c). This shows that the observed correlations cannot be reproduced by classical light.

SUPPLEMENTARY REFERENCES

[1] Barak, R. & Ben-Aryeh, Y., Quantum fast Fourier transform and quantum computation by linear optics, *J. Opt. Soc. Am. B* **24**, 231–240, (2007).
[2] Laing, A. & O'Brien, J. L., Super-stable tomography of any linear optical device, Preprint arXiv:1208.2868v1 (2012).
[3] Shchesnovich V. S., Partial indistinguishability theory for multiphoton experiments in multiport devices, *Phys. Rev. A* **91**, 013844 (2015).
[4] Tichy M.C., Sampling of partially distinguishable bosons and the relation to the multidimensional permanent, *Phys. Rev. A* **91**, 022316 (2015).
[5] Tillmann M., Tan S. H., Stoeckl S.E., Sanders B.C., De Guise H., Heilmann R., Nolte S., Szameit A. & Walther P., Generalized Multiphoton Quantum Interference, *Phys. Rev. X* **5**, 041015 (2015).
[6] Arkhipov A., Boson sampling is robust to small errors in the network matrix, arXiv:1412.2516v1 [quant-ph] (2014).
[7] Shchesnovich V. S., Tight bound on the trace distance between a realistic device with partially indistinguishable bosons and the ideal BosonSampling, *Phys. Rev. A* **91**, 063842 (2015).
[8] Bromberg Y., Lahini Y., Morandotti R. & Silberberg Y., Quantum and Classical Correlations in Waveguide Lattices, *Phys. Rev. Lett.* **102**, 253904 (2009).
[9] Peruzzo A., Lobino M., Matthews J.C.F., Matsuda N., Politi A., Poulios K., Zhou X.-Q., Lahini Y., Ismail N., Wörhoff K., Bromberg Y., Silberberg Y., Thompson M.G. & O'Brien J.L., *Science* **329**, 1550-1503 (2010).
